# Promoted Viability and Differentiated Phenotype of Cultured Chondrocytes With Low Level Laser Irradiation Potentiate Efficacious Cells for Therapeutics

**DOI:** 10.3389/fbioe.2020.00468

**Published:** 2020-05-29

**Authors:** Xiaohong Yang, Timon Chengyi Liu, Shaojie Liu, Weicong Zhu, Honglin Li, Peihong Liang, Suihui Ye, Shuliang Cui

**Affiliations:** ^1^Guangzhou Institute of Traumatic Surgery, Guangzhou Red Cross Hospital, School of Medicine, Jinan University, Guangzhou, China; ^2^Laboratory of Laser Sports Medicine, College of Physical Education and Sports Medicine, South China Normal University, Guangzhou, China; ^3^Surgical Department, Guangzhou Red Cross Hospital, School of Medicine, Jinan University, Guangzhou, China; ^4^School of BioSciences, The University of Melbourne, Melbourne, VIC, Australia

**Keywords:** low level laser irradiation (LLLI), chondrocyte viability, anti-apoptosis, chondrogenic phenotype, effective chondrocytes for tissue engineering

## Abstract

Effective clinical treatments of cartilage lesions in affected joints require large numbers of viable chondrogenic cells generated through *in vivo* stimulation or *ex vivo* expansion of chondrocytes isolated from small biopsy specimens. Conventional passaging of chondrocytes in culture provides sufficient cells for treatments but these cells usually lose their differentiated phenotype. This leads to the formation of fibrocartilaginous tissue due to a malfunctioning repair process. Biostimulation of passaging chondrocytes with low level laser irradiation (LLLI) may theoretically produce more functional chondrocytes for cell-based repair of cartilage defects. Molecular and cellular analyses, cytochemistry, cell cultivation, and microscopy showed that LLLI treatments were found to (1) increase chondrocyte viability, (2) promote secretion of matrix proteins, (3) upregulate expression of chondrogenic genes, and (4) downregulate gene expression of cell destructive proteases and genes coding for mediators involved in the extrinsic apoptosis signaling pathway. Furthermore, LLLI attenuated induction of genes associated with cell death and matrix breakdown induced by IL-1β, some of which was seen at the protein level, with verification of effects on gene expression in the C28/I2 human chondrocyte line. LLLI treatments during culture generated larger numbers of viable chondrocytes compared to untreated cultures. Moreover, LLLI-treated chondrocytes in culture also rectified and simultaneously maintained their differentiated phenotype. Cultured chondrocytes treated with LLLI are a promising cell source for repairing cartilage lesions *in vivo* and restoration of articular function using tissue engineering strategies.

## Introduction

The ability of articular cartilage for remodeling and damage repair is limited by its avascular nature, and by the low cellularity (Oldershaw, 2012) and functionality (Archer and Francis-West, 2003) of chondrocytes in surface layers. To date, effective clinical treatments for repairing cartilage defects include autologous chondrocyte implantation (ACI) (Brittberg et al., 1994), matrix-induced autologous chondrocyte implantation (MACI) (Gibson et al., 2006), microfracture (Steadman et al., 1999) and allografts (Gortz and Bugbee, 2007), all of which depend upon the availability of viable chondrocytes. A large number of functional cells are usually achieved by *ex vivo* expansion of chondrocytes isolated from a small biopsy cartilage specimen and expanded through at least four passages (Darling and Athanasiou, 2005). However, a plethora of evidence showed that passaged chondrocytes alter their gene expression profiles (Lin et al., 2008) and become more fibroblastic (Stokes et al., 2001). This process of dedifferentiation typically shows decreased collagen type II (COL II) and aggrecan (ACAN) accompanied by increased collagen type I (COL I) (Hsu et al., 2002; Darling and Athanasiou, 2005; Frohlich et al., 2007). Dedifferentiated chondrocytes have failed to achieve long term repair and restoration of functional articular cartilage due to the formation of fibrocartilage as shown in ACI and MACI (Roberts et al., 2009), and microfracture (Gobbi et al., 2005). Effective numbers of expanded chondrocytes with enhanced differentiated phenotype could be achieved by modulation with various factors, including the approach of easily accessible laser irradiation.

Low level laser therapy (LLLT) has been used widely in a variety of biomedical treatments based on its modulatory effect on cell growth and metabolism through photobiostimulation, which permeabilizes the membrane to allow physiological changes in target cells (Pinheiro et al., 2002). The photons enter the cell and are readily absorbed by a photoreceptor leading to the photoactivation of target molecules for bioreactions or signal transduction (Smith, 1991; Karu, 1998) to enhance cell proliferation and function. Low doses of laser irradiation increase cytoplasmic Ca^2+^ to stimulate various biological processes. Higher doses release too much Ca^2+^ for the ATPase-powered calcium pumps, severely depleting cellular energy so that cell metabolism is compromised (Smith, 1991; Schindl et al., 2000). The LLLT-treated target cells respond with increasing cellular activity to increasing doses until a peak is reached. Higher doses then result in decreasing cellular responses in a “biphasic dose response” pattern (Alghamdi et al., 2012). All LLLT treatments are pursuing an optimal threshold of irradiation regime for maximal biostimulation of the target cells.

Early attempts of determining the effect of laser radiation on chondrocytes applied various wavelengths, power intensities and exposure periods in LLLT. Low doses of LLLT treatments showed retention of chondrocyte viability that was reduced with higher doses in nasal septal cartilage specimens (Rasouli et al., 2003); activated DNA synthesis in regenerating chondrocytes surrounded the LLLT spots (Wong et al., 2005), which restricted its effect on collagen type II (COL II) but not on COL I (Holden et al., 2009). To enhance chondrogenesis, low level blue laser (405 nm, 100 mW/cm^2^) stimulated the expression of chondrogenic genes in prechondrogenic ATDC5 cells (Kushibiki et al., 2010). The use of a red laser (780 nm, 2500 mW) promoted viability and cell metabolism in cultured human chondrocytes (Morrone et al., 2000), and similar laser treatments increased and maintained proliferation of cultured rabbit and human chondrocytes (Torricelli et al., 2001). Low level red light irradiation (658, 785, and 830 nm with 10–70 mJ/cm^2^) increased proliferation in chondrocytes cultured in medium with 2 and 5% PBS, but not with 0 and 10% FBS (Zheng et al., 2015). However, most experimental evidence using cultured chondrocytes show cell viability results by MTT/MTS assays with few chondrogenic genes examined for expression. Based on the above investigations, we hypothesized that low level laser irradiation (LLLI) would effectively modulate the proliferation and differentiation of chondrocytes in culture for promoted viability and enhanced differentiated phenotype. We have adapted some culture conditions, such as FBS concentrations in the medium, and employed a LLLI of He-Ne laser (632.8 nm) to stimulate chondrocyte growth, aiming at producing large numbers of chondrocytes with rectified phenotype *ex vivo*. Our findings are that LLLI significantly increased proliferation of cultured chondrocytes, upregulated expression of chondrogenic genes, and downregulated expression of genes involved in the apoptotic signaling pathway and cell destruction, all of which contributed to the generation of passaged chondrocytes with strengthened chondrogenic properties applicable to tissue engineering. The results of the current study implicate a modulatory mechanism by LLLI for chondrocytic phenotype *in vitro*. The passaged chondrocytes with high viability and an enhanced differentiated phenotype may represent an efficacious cell source for repair of cartilage lesions in cell-based therapies.

## Materials and Methods

### Isolation and Culture of Primary Chondrocytes

Fresh cartilage samples were collected from 3-week-old New Zealand white rabbits under the guidance of the governing authority of Guangzhou Red Cross Hospital, Jinan University School of Medicine (SHYYLS 2017-004-01 and SHYYLS 2019-040-01). The rabbits were used for isolation of chondrocytes as previously described (Yang et al., 2017). Briefly, the chondrocytes were isolated by sequential enzymatic digestions and harvested for monolayer culture in Dulbecco’s Modified Eagle Medium: Nutrient Mixture F-12 (DMEM-F12, GIBCO/Life Technology, Grand Island, NY, United States) containing 10% fetal bovine serum (FBS, GIBCO) and 1% penicillin-streptomycin (GIBCO) at 37°C with 5% CO_2_ in the *Forma STERI-CYCLE CO_2_* incubator (Thermo Scientific, Marietta, OH, United States). About 8 × 10^5^/ml cells were seeded in 25 cm^2^ flasks and cultured to ≥80% confluence and then propagated to obtain passage 3/4 chondrocytes for experiments.

### Design of Laser Irradiation Scheme

Laser irradiation was applied to stimulate chondrocytes in culture for modulation of their growth and cellular phenotype. The chondrocytes were treated by LLLI using the HN-8318 He-Ne Laser Radiator (Guangzhou Institute of Laser Technology and Application, Guangzhou, China) with an optical output power of 12 mW over 0.91 cm^2^ projected to the cells growing in a culture plate with a capacity required by different experiments. Chondrocytes treated by this output intensity for a duration of 1, 3, 5, 8, 11, and 13 min daily for 1 and 3 days, respectively, were examined for simulated cellular viability by 3-(4,5-dimethylthiazol-2-yl)-5-(3-carboxymethoxyphenyl)-2-(4-sulfophenyl)-2*H*-tetrazolium) assay (MTS). The chondrocytes treated with LLLI for 8 min achieved the best cellular viability in both 1 day and 3 day treatments, then the “12 mW over 0.91 cm^2^ for 8 min” was calculated to give rise of 5.74 J/cm^2^ as an intensity unit and used in the further experiments. The application of the intensity unit was described as an “LLLI of 5.74 J/cm^2^ daily in culture for 1 or 3, or 5 day(s)” in this study.

### MTS Assay to Detect Proliferation of Chondrocytes

Aliquots of 8 × 10^3^ cells/cm^2^ were seeded in the wells of a 48-well plate and cultured in DMEM-F12 medium supplemented with 2.5% FBS overnight before treated for 1, 3, 5, 8, 11, and 13 min with output power of 12 mW over 0.91 cm^2^ daily for 1 day or 3 days followed by further 24 h culture, then assayed for cellular viability by MTS using *CellTiter 96*^®^ *AQueous One Solution Cell Proliferation Assay* kit according to the manufacturer’s instruction (Promega, Madison, WI, United States) to measure OD_490_ values with the GloMax Multi Detection System (Promega).

### Glycosaminoglycan Released by Cultured Chondrocytes

Matrix protein glycosaminoglycans (GAGs) released to the medium by cultured rabbit chondrocytes were analyzed using an Alcian blue assay for specific detection of sulfated GAGs (Frazier et al., 2008). Aliquots of 3.6 × 10^3^ rabbit cells/cm^2^ were cultured in a 12-well plate overnight in DMEM-F12 medium complemented with 2.5% FBS, then treated by LLLI of 5.74 J/cm^2^ daily in culture for 6 days followed by further cultivation for 6 days. The GAGs in medium collected on days 2, 4, 6, 8, 10, and 12 were stained with Alcian Blue 8GX (Sigma-Aldrich, Saint Louis, MO, United States). The samples and a standard solution containing 100, 200, 400, 600, 800, and 1,000 μg/ml of chondroitin sulfate (Sigma-Aldrich) were mixed with fresh dye solution and incubated at 37°C for 10 min. An aliquot of 150 μl sample (*n* = 4) was mixed with 2.25 ml of dye solution (H_2_O as blank) for spectrophotometric measurement of OD_480_ values, which were used for calculation of GAG concentrations in samples (μg/ml).

### High-Density Micromass Cultures for Matrix Deposition in Cultured Chondrocytes

The accumulated matrix deposited in the cultured chondrocytes was assayed by micromass culture combined with the staining of matrix protein proteoglycan (largely glycosaminoglycan) as a marker (Schlichting et al., 2014). The high-density micromass culture was initiated by seeding 6.4 × 10^6^ cells/cm^2^ (P3/4 rabbit chondrocytes) in wells of a 24-well plate, and cultured in an incubator with 5% CO_2_ supply at 37°C for 2 h, then supplied with 1 ml of DMEM-F12 medium containing 5% FBS followed by further 24 h incubation. The chondrocytes were allocated to an untreated control and 3 groups were treated by LLLI of 5.74 J/cm^2^ daily in culture for 1, 3, and 5 days, respectively. The culture medium was changed every second day. The cell masses were harvested and fixed for 30 min in 0.5 ml/well of paraformaldehyde, washed 3 times in distilled water, and then specifically stained for sulfated GAGs with 0.1% solution of Safranin O (Sigma-Aldrich) (Király et al., 1996), for 10 min followed by 3× quick washes before observation under a stereo microscope (Nikon SMZ 25, Nikon Corporation, Minato-ku, Tokyo, Japan) with a 1.8× magnification to acquire digital images.

### Scanning Electron Microscopy

Aliquots of 2.8 × 10^2^ rabbit cells/cm^2^ were cultured on 24 × 24 mm coverslips pre-coated with poly-lysine overnight. The chondrocytes were treated by LLLI of 5.74 J/cm^2^ daily in culture for 6 days followed by further culture for 6 days with the medium changed every second day. Cells in culture were harvested at day 5 and day 9 for microscopic assessment. The chondrocytes on coverslips were fixed in 2.5% glutaraldehyde prepared in 1 X PBS for 24 h and then examined using scanning electron microscopy (SEM, XL-30-based Environmental Scanning Electron Microscope, Philips, Hilversum, Netherlands) for assessment of detailed effects of LLLI on their growth.

### Nucleic Acid Labeling of Chondrocytes by Acridine Orange

The metachromatic compound acridine orange (AO) was used as a nucleic acid-selective fluorescent cationic dye for assessing synthetic activity in cycling cells. AO penetrates the cell membrane and interacts with DNA by intercalation, showing spectrally similar to fluorescein as in green. When AO associates with RNA by electrostatic attractions, it emits fluorescent red (Darzynkiewicz and Huang, 2004). The images of AO-labeled DNAs and RNAs were acquired using microscopy (Li et al., 2015; Byvaltsev et al., 2019) and quantitatively analyzed using the density of labeled nucleic acids (Parng et al., 2004; Yang et al., 2017). Aliquots of 2.8 × 10^2^ rabbit cells/cm^2^ (P3/4 chondrocytes) were plated on 24 × 24 mm coverslips pre-coated with poly-lysine and cultured overnight prior to treatment with LLLI of 5.74 J/cm^2^ daily in culture for 6 days. The chondrocytes on coverslips were washed and stained for 10 min with 0.01% acridine orange (Sigma-Aldrich) to selectively label the DNA and RNA. After extensive washing, the stained cells were analyzed by confocal laser scanning microscopy (CLSM) using the *Zeiss LSM 510 META System* (Carl Zeiss Advanced Imaging Microscopy, Jena, Germany) under unique setups (parameters) for each group. Images were acquired by dual channels of green for DNA (excitation at 488 nm; emission at 520 nm) and red for RNA (excitation at 453 nm; emission at 615 nm). The density of labeled DNA and RNA was quantified using the Zeiss Physiology/TimeSeries for Release 3.2 as levels of DNA and RNA in the cell content. The ratio of the intensity in LLLI-treated cells over that in untreated cells was used as an indicator for LLLI modulation of cellular synthesis.

### Western Blot Analysis for Protein Expression in Cultured Chondrocytes

Aliquots of 4.2 × 10^3^ rabbit cells/cm^2^ were cultured in a 6-well plate overnight, then treated by LLLI of 5.74 J/cm^2^ daily in culture for 1, 3, and 5 days. Harvested chondrocytes were resuspended in RIPA Lysis and Extraction Buffer (Thermo Scientific), containing 25 mM Tris-HCl pH 7.6, 150 mM NaCl, 1% NP-40, 1% sodium deoxycholate, 0.1% SDS with 1 mM PMSF, and incubated on ice before sonicated using Sonics Vibra Cell^TM^ (Sonics & Materials Inc., Newtown, CT, United States) for 25 s. The lysates were centrifuged at 13,000 × *g* for 10 min at 4°C and supernatants harvested for protein analysis.

Total proteins were separated by sodium dodecyl sulfate–polyacrylamide gel electrophoresis (SDS-PAGE), then transferred onto polyvinylidene difluoride (PVDF) membranes (88520, Pierce Biotechnology, Rockford, IL, United States) by Western blotting as described previously (Yang et al., 2017). Briefly, membranes were blocked in TBST buffer in 5% fat-free milk powder solution, pH 7.4, then incubated with diluted primary antibodies for COL2A1 and COL1A (Santa Cruz), Anti-CASP-3 (p17) antibody (BA2885-2, Boster Biological Technology, Wuhan, China), and rabbit MMP13 antibody (BA0574, Boster), respectively. The monoclonal Anti-β-Actin antibody (Sigma-Aldrich) against cytoskeletal protein β-actin was used as an internal control. Binding proteins were detected with goat anti-rabbit IgG-HRP conjugate (Santa Cruz), visualized using Clarity^TM^ Western ECL Substrate Kit (Bio-Rad), and analyzed by the computer software Image Lab (Beta 1) Version 3.0.1 (Bio-Rad).

### Quantitative Real Time PCR (qRT-PCR) Detection of Gene Expression

Rabbit chondrocytes were treated in culture as for Western blot. The immortalized human chondrocyte cell line, C28/I2, was employed for verification of gene expression. Human C28/I2 cells strongly express the chondrogenic master transcription factor gene Sox9, which, in turn, regulates the chondrocytic phenotype to express high levels of matrix-anabolic and matrix-catabolic genes suitable for investigation of regulatory mechanism in chondrocyte metabolism (Finger et al., 2003; Claassen et al., 2011). C28/I2 chondrocytes enables the assessment of the relationship of cellular and molecular findings to the physiologic function. However, these cells lack expression of genes involved in matrix synthesis and turnover with the process of immortalization favoring proliferation and cycling (Finger et al., 2003).

For both rabbit and human chondrocytes, an aliquot of 1 × 10^4^ cells/cm^2^ was cultured in a 6-well plate for 24 h, then treated by LLLI as for rabbit chondrocytes without IL-1β stimulation. Total RNA was isolated and purified from the chondrocytes lysed using TRIzol reagent (Invitrogen, Guangzhou, China) and quantified using NanoDrop ND-1000 v3.3.0 (Thermo Fisher Scientific, Wilmington, DE, United States). One microgram of total RNA were reversely transcribed using RNA transcriptase (TaKaRa, Shiga, Japan) and quantified by qRT-PCR using the All-in-One qPCR Mix (GeneCopoeia, Rockville, MD, United States) in 96-well microplates by thermal cycling of 95°C for 2 min followed by 40 cycles at 95°C for 5 s and 60°C for 15 s. Untreated chondrocytes (LLLI^–^ or IL-1β^–^) were compared to their treated counterparts (LLLI^+^ and/or IL-1β^+^) and the expression level in each sample (*n* = 3) was normalized against the internal reference gene of glyceraldehyde 3-phosphate dehydrogenase (*GAPDH*) by the 2^–ΔΔ*CT*^ comparative method established previously (Livak and Schmittgen, 2001) using “control (LLLI^–^) chondrocytes cultured for 1 day” as a calibrator in each group. The expression of genes involved in chondrocyte growth was investigated in rabbit cells cultured in monolayer, including the category 1 genes involved in cell growth/proliferation and phenotypic property definition and category 2 genes coding for regulators/mediators of cell apoptosis pathways and matrix degradation. Selected regulatory genes encoding transcription factors, cytokines, and proteases (from both categories) were also examined for verification of LLLI-regulated expression in cultured human C28/I2 chondrocytes. Information for genes assessed in this study is given in Supplementary Table S1.

### Live/Dead Staining Assay Using Double Fluorescent Dyes in Cultured Chondrocytes

The effect of LLLI on growth of cultured chondrocytes was examined by live/dead staining using DiO (3,3′-dioctadecyloxacarbocyanine, perchlorate) and PI (propidium iodine) double labeling. DiO is a green fluorescent, lipophilic carbocyanine dye binding to membrane/cell surface of live cells and the nuclei staining dye PI cannot pass through the membrane of viable cells, but stains DNA when reaches through disordered areas of dead cell membrane to emit red fluorescence. About 2.1 × 10^4^ rabbit cells/cm^2^ were cultured in a 6-well plate containing the DMEM-F-12 medium in an incubator with 5% CO_2_ supply at 37°C for 24 h followed by LLLI of 5.74 J/cm^2^ daily in culture for 5 days. Medium was changed every second day. The treated and untreated control cells were stained with 4 μM DiO (C1038, Beyotime Biotechnology, Haimen, Jiangsu, China) solution in DMEM-F12 medium without FBS by incubation at 37°C for 25 min. The stained cells were then washed in 1 X PBS twice and further stained in 1.48 nM PI (P4107, Sigma-Aldrich) solution by incubation at 37°C for 5 min. Images of stained cells were viewed and captured using CLSM (Zeiss LSM 510 META System). The labeling signals in the chondrocytes were acquired by the dual channels with CLSM and quantitatively analyzed using the Zeiss Physiology/TimeSeries for Release 3.2. The green/red ratio in treated and control cells was used to indicate changes of live/dead cells.

### Statistical Analysis

Data was collected from ≥3 independent experiments, means of which were used for analysis using the SPSS 22 (IBM SPSS Statistics, Chicago, IL, United States). Differences between experiments and their untreated control counterparts were determined by One-way ANOVA and presented as mean ± standard deviation (bars). The Cochran-Armitage test for trend was used to assess differences of GAG levels secreted by cultured chondrocytes at various time points and analyze the trend of variance. Differences with a *P* < 0.05 (^∗^) or *P* < 0.01 (^∗∗^) value show statistical significance at the respective confidence level.

## Results

### Modulation of Viability by LLLI in Cultured Chondrocytes

**FIGURE 1:**
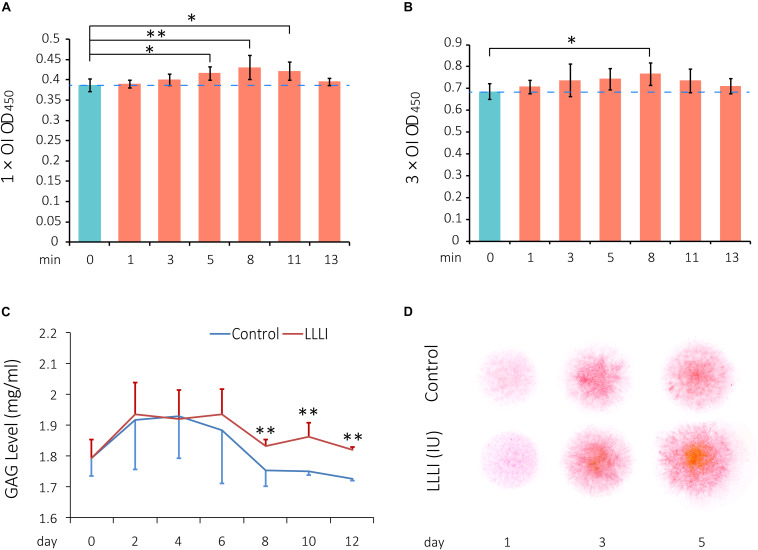
Cell viability assay of LLLI-stimulated rabbit chondrocytes by MTS, measurement of released glycosaminoglycan (GAGs), and matrix deposition in micromass culture. **(A)** Chondrocytes were treated with the output intensity of LLLI for various time periods and cultured for 24 h before assay by MTS. **(B)** Chondrocytes were treated as **(A)** but with the output intensity daily in culture for 3 days and then analyzed by MTS. The figure was generated using data from triplicate samples, each of which were experimentally examined in triplicate. **(C)** GAGs released in the culture medium by the chondrocytes was measured using Alcian blue assays. The cells treated with LLLI of 5.74 J/cm^2^ daily in culture showed no difference in GAG levels within the treatment period (0–6 days). Thereafter, GAG in LLLI-treated cells (red) maintained significantly higher levels than that in control cells (blue) in the post-treatment period (8–12 days) with a different trend. **(D)** Chondrocytes in micromass were treated with LLLI of 5.74 J/cm^2^ daily in culture for 1, 3, and 5 days and changes in matrix deposition were detected by Safranin O staining, which showed an enhancement in a time- and LLLI dosage-dependent manner. Results are the mean ± SEM (*n* = 4). OI = output power of 12 mW over 0.91 cm^2^. Error bar = SD. ^∗^*P* < 0.05; ^∗∗^*P* < 0.01.

To investigate the effect of LLLI on regulation of viability/proliferation, rabbit chondrocytes were allocated to two experimental groups and treated by LLLI with an output power of 12 mW over 0.91 cm^2^ in a time course daily for 1 and 3 days, respectively, followed by further culture for 24 h prior to harvest for MTS assays. The viability of cultured chondrocytes treated by LLLI for 5 min (3.58 J/cm^2^) in the 1 day treatment group became significantly (*P* < 0.05) higher than that of untreated cells, peaked (*P* < 0.01) at 8 min (5.74 J/cm^2^), then decreased to a significant level as treated for 11 min (7.87 J/cm^2^) before becoming insignificant when treated for 13 min (9.30 J/cm^2^) (Figure 1A). Cultured chondrocytes responded to LLLI treatment for 3 days with a significant increment (*P* < 0.05) in cellular viability by 8 min (≈17 J/cm^2^) (Figure 1B). Compared to the respective control group, the highest cellular viability was achieved by stimulation for 8 min in both LLLI treatment groups.

### Effect of LLLI on Glycosaminoglycan Secretion and Matrix Deposition by Cultured Chondrocytes

Glycosaminoglycans (GAGs) synthesized and released to the medium by cultured chondrocytes were measured by Alcian blue colorimetric assay. Both LLLI-treated and untreated control groups used the same starting numbers of cells (3.6 × 10^3^ rabbit cells/cm^2^) under identical culture conditions with changes in GAG levels implicated overall LLLI modulation. The effect of LLLI treatment on secretion of GAGs in cultured chondrocytes exhibited a biphasic pattern as revealed by a two-factor repeated measurement data analysis of variance-trend (Cochran Armitage test for trend). There was no difference in GAG secretion levels between LLLI-treated chondrocytes and the untreated control (*P* > 0.05) during treatment (day 0 to day 6). The LLLI-treated cells exhibited significantly higher (*P* < 0.01) secretion levels than the untreated control in the post-treatment period (day 8 to day 12) (Figure 1C). The two phases follow a similar trend, with LLLI-treated chondrocytes with high matrix secretion diverging from trend post-treatment upon visual inspection.

The rabbit chondrocytes treated with LLLI of 5.74 J/cm^2^ daily in culture for 1, 3, and 5 days and their untreated counterparts formed semi-spherical micromasses with LLLI-treatment resulting in the formation of larger cell masses and increased matrix deposition as detected by Safranin O staining for sulfated proteoglycans (Figure 1D).

### Evidence of LLLI-Enhanced Chondrocyte Morphology by SEM

**FIGURE 2:**
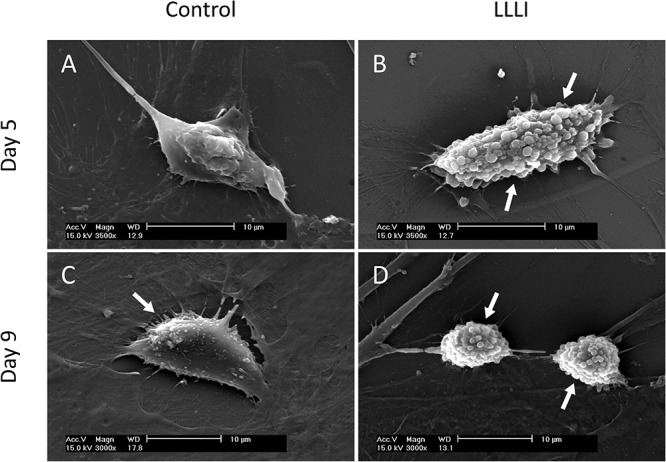
Sub-cellular analysis of rabbit chondrocytes by SEM. **(A)** Chondrocytes cultured in monolayer for 5 days only (control); **(B)** Chondrocytes cultured in monolayer treated with LLLI of 5.74 J/cm^2^ daily in culture for 5 days; **(C)** Chondrocytes cultured in monolayer for 9 days only (control); **(D)** Chondrocytes were treated with LLLI of 5.74 J/cm^2^ daily in culture for 6 days followed by further culture up to day 9. “Arrows” indicates secretory granules on the cell surface. Scale bar (—) represents 10 μm.

SEM images exhibited the typical morphology of cultured rabbit chondrocytes. Chondrocytes were treated by LLLI of 5.74 J/cm^2^ daily in culture for 5 and 6 days, followed by further culture up to 9 days with accumulated intensities of 28.7 and 34.44 J/cm^2^, respectively. Cell morphology was examined at day 5 and day 9. The control cells exhibited a flattened elongated morphology spread along the surface (Figure 2A), but the LLLI-treated chondrocytes with 28.7 J/cm^2^ showed relatively spindle-shaped, spherical cells loosely adhered to the plastic surface (Figure 2B). Chondrocytes in culture for 9 days without LLLI treatment evolved into a polygonal shape with few secretory granules (arrows) isolated by irregular lacunae-like spaces (Figure 2C) while after treatment with LLLI of 5.74 J/cm^2^ daily in culture for 6 days (34.44 J/cm^2^) and cultured until day 9, the chondrocytes exhibited an ovoid morphology with many secretory granules on the surface (arrows), implying an active state (Figure 2D).

### Cytomorphological Evidence of LLLI Effect on Anabolic Synthesis in Cultured Chondrocytes

**FIGURE 3:**
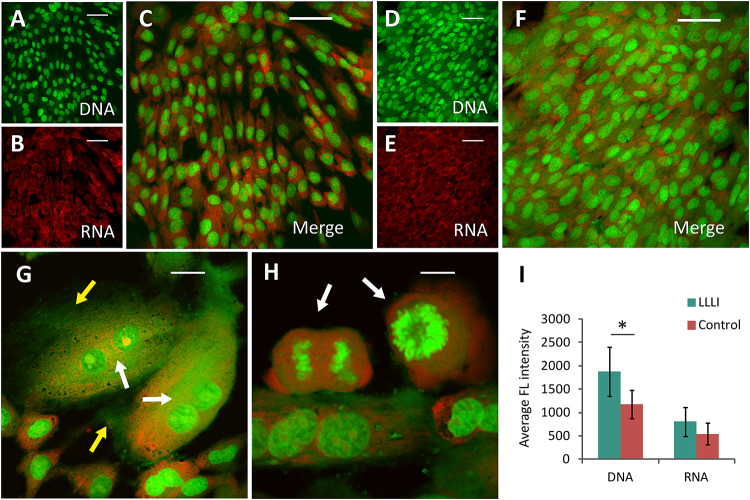
Cytomorphological analysis of cultured rabbit chondrocytes by acridine orange staining and CLSM. The differentially labeled DNA and RNA in cells excite and emit at different wavelengths as fluorescent green and red, respectively. The dual labeling unveils the cells and their relative intensity implicative of cellular activity as DNA is duplicated and RNA synthesized. **(A–C)**, images of untreated chondrocytes with stained DNA **(A)** and RNA **(B)** merged into dual-channel image **(C)** acquired by CLSM. **(D–F)** images of chondrocytes treated by LLLI of 5.74 J/cm^2^ daily in culture for 6 days, stained for DNA **(D)** and RNA **(E)**, and the merged image **(F)**. **(G,H)** enhanced anabolic synthesis in LLLI-treated chondrocytes shown as typical multi-shaped cells with double- and poly-nuclei cells. Duplicating and mitotic chromosomes and cell aggregates are indicted by arrows. **(I)** significant higher levels of DNA (green) and RNA (red) in LLLI-treated chondrocytes than untreated cells as shown by quantification of the dual fluorescent labeling. Scale bar (—) represents 50 μm. Daily LLLI treatment = 5.74 J/cm^2^ of LLLI for 8 min; Error bar = SD; ^∗^*P* < 0.05.

In cultured chondrocytes, AO entered the cellular compartments resulting in red fluorescent staining of RNA with labeled DNA in the nucleus in bright green. Based on the unique spectral distinctions and differential staining of RNA and DNA molecules, the level of synthetic activity of nucleic acids in the cultured chondrocytes was distinguishable in different cell populations at a given stage, which was quantitatively analyzed using CLSM (Yang et al., 2017). The nucleic acid staining of LLLI-treated and untreated control rabbit chondrocytes showed clearly outlined ovoid- to spindle-shaped nuclei (DNA in green fluorescence) in untreated cells (Figures 3A,C), but LLLI-treated cells synthesized more DNA (Figures 3D,F). Untreated chondrocytes showed some red-labeled clumps of irregular nuclear RNA with acidified compartments around the nuclei (Figures 3B,C). The LLLI-treated chondrocytes possess cellular characteristics of active anabolism shown as more DNA synthesis (greener labeled DNA) and typical multi-shaped cells with occasional, double-, and poly-nucleated cells, duplicating and mitotic chromosomes and cell aggregates (white arrows) (Figures 3G,H) under CLSM. Quantitative analysis of labeled nucleic acid revealed significantly (*P* < 0.05) higher DNA levels in LLLI-treated compared to untreated chondrocytes with a trend of higher RNA content (Figure 3I).

### Effect of LLLI Treatment on Protein Expression in Cultured Chondrocytes

**FIGURE 4:**
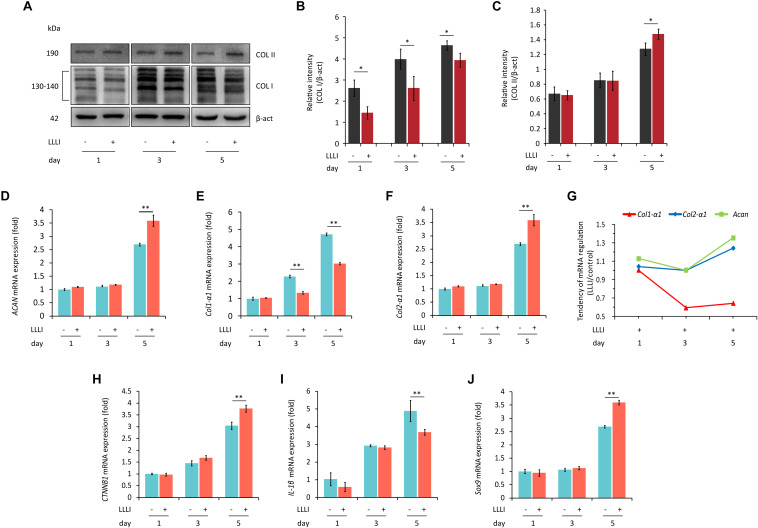
Western blots and qRT-PCR analysis for gene expression in cultured rabbit chondrocytes. Cells were treated with LLLI of 5.74 J/cm^2^ daily in culture for 1, 3, and 5 days, respectively, followed by further culture for 24 h before harvesting for preparation of total protein and total RNA samples (*n* = 3). **(A–C)** Western blot analysis using the COL1A and COL2A1 antibodies showing reduced “fibroblastic matrix protein” COL I in response to all 3 treatments of LLLI (^∗^), while the “chondrogenic matrix protein” COL II remained at similar levels in treated and untreated cells after exposure to LLLI of 5.74 J/cm^2^ daily in culture for 1 and 3 days, but was increased by LLLI of 5.74 J/cm^2^ daily in culture for 5 days (^∗^), against the internal control protein β-actin (β-act). **(D–J)** qRT-PCR analysis of gene transcription in chondrocytes. Unchanged expression of *Acan*, *Col2-*α*1*, *CTNNB1*, *IL-1*β, and *Sox9* in chondrocytes untreated or treated with LLLI of 5.74 J/cm^2^ daily in culture for 1 and 3 days **(D–J)** with significantly decreased (^∗∗^) *Col1-*α*1* **(E)** expression in cells treated with LLLI of 5.74 J/cm^2^ daily in culture for 3 days. When treated by LLLI of 5.74 J/cm^2^ daily in culture for 5 days, the expression of all 6 genes were highly (^∗∗^) modulated in cultured chondrocytes, among which, *Acan* **(D)**, *Col2-*α*1* **(F)**, *CTNNB1* **(H)**, and *Sox9* **(J)** were significantly (^∗∗^) increased while *Col1-*α*1* **(E)** and *IL-1*β **(I)** were significantly (^∗∗^) reduced. The pattern of upregulated *Col2-*α*1* and *Acan* with downregulated *Col1-*α*1* as plotted using the ratio of LLLI/control amplicons represents an ameliorated differentiated phenotype **(G)**. The β-actin protein and *GAPDH* gene were used as internal references, respectively. Daily LLLI treatment = 5.74 J/cm^2^ of LLLI for 8 min; Error bar = SD. ^∗^*P* < 0.05 and ^∗∗^*P* < 0.01.

**FIGURE 5:**
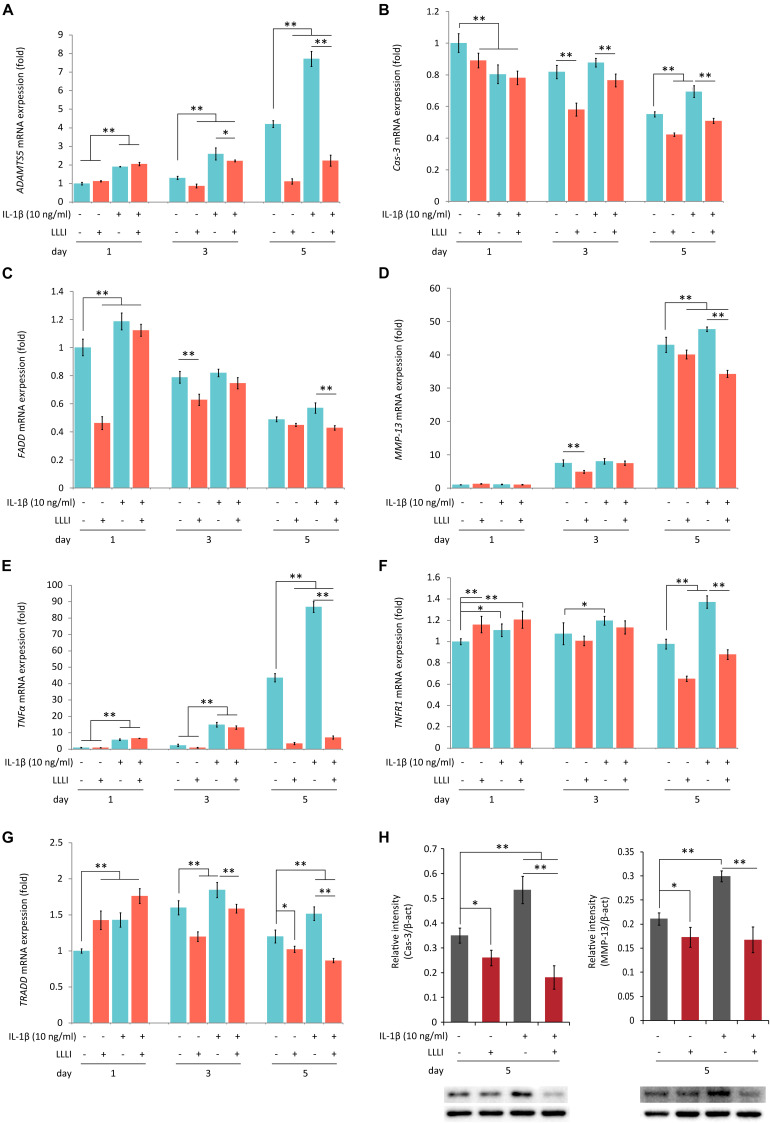
Protein Western blots and qRT-PCR analysis of the expression of genes associated with cell death and matrix destruction in rabbit chondrocytes. The cells were treated with pro-inflammatory cytokine IL-1β, and LLLI of 5.74 J/cm^2^ daily in culture for 1, 3, and 5 days, respectively, or simultaneously treated with IL-1β, followed by further culture for 24 h before harvested for isolating total RNA (*n* = 3). The comprehensive review of LLLI-regulated expression of the genes revealed 3 major patterns: (1) the “destructive” genes expressed at low basal level under the influence of treatment with LLLI of 5.74 J/cm^2^ daily in culture for 1 day, such as *ADAMTS5* **(A)**, *MMP-13* **(B)**, and *TNF-*α **(E)**; (2) differentially expressed cell death mediator genes at a high basal level, such as *Cas-3* **(B)**, *FADD* **(C)**, *TNFR1* **(F)**, and *TRADD* **(G)**; (3) treated by LLLI of 5.74 J/cm^2^ daily in culture for 3 days, the IL-1β-induced expression of *ADAMTS5* **(A)** and *Cas-3* **(B)** were downregulated. The LLLI of 5.74 J/cm^2^ daily in culture for 5 days decreased the expression of genes involved except *FADD* **(C)**; all the genes were highly increased by IL-1β, then counteracted by the LLLI of 5.74 J/cm^2^ daily in culture for 5 days. The *GAPDH* was used as an internal reference. The altered protein expression associated with LLLI-modulated gene expression were carried out by Western blot analysis, which showed significantly decreased Cas-3 (**H**, left) and MMP-13 (**H**, right) by LLLI of 5.74 J/cm^2^ daily in culture for 5 days. The charts were plotted using data from triplicated samples, each of which was experimented in triplicates. Daily LLLI treatment = 5.74 J/cm^2^ of LLLI for 8 min; Error bar = SD; ^∗^*P* < 0.05 and ^∗∗^*P* < 0.01.

To examine the protein expression and verify the modulation of LLLI treatment at a protein level in cultured rabbit chondrocytes, the phenotype-illustrative matrix proteins (COL I and COL II) and cell destructive proteases, caspase-3 (Cas-3), and matrix metalloproteinase 13 (MMP-13) were detected using the cytoskeleton protein β-actin as an internal reference control. COL I and COL II protein were detected by Western blotting (Figure 4A). COL I was significantly downregulated by all 3 doses of LLLI treatments (*P* < 0.05) (Figure 4B) while the level of the COL II protein in chondrocytes treated by LLLI of 5.74 J/cm^2^ in culture for 1 and 3 days remained unchanged (Figure 4C). Furthermore, chondrocytes treated by LLLI of 5.74 J/cm^2^ daily in culture for 5 days expressed a significantly higher level (*P* < 0.05) of COL II than that in untreated control cells (Figure 4C). The protein level of Cas-3 and MMP-13 in cultured chondrocytes was significantly reduced by LLLI of 5.74 J/cm^2^ in culture for 5 days (*P* < 0.05), highly upregulated by IL-1β treatment (*P* < 0.01) which was strongly offset by the LLLI treatment (*P* < 0.01) (Figure 5H).

### Effect of LLLI on the Expression of Genes Involved in Chondrocyte Growth and Differentiated Phenotype

Based on the observation that the viability of cultured rabbit chondrocytes was strengthened by LLLI (Figure 1), the expression of genes from two categories was further investigated, including genes involved in cell growth/proliferation and phenotypic property defining, and genes encoding regulators/mediators of cell death cascade and matrix degradation (see Supplementary Table S1 for details of designed primers and their amplicons).

Category 1: Genes related to cell growth/proliferation and chondrocyte phenotype. qRT-PCR results showed that the level of gene expression of *Acan*, *Col2-*α*1*, *CTNNB1*, *IL-1*β, and *Sox9* remained unchanged in chondrocytes treated with LLLI of 5.74 J/cm^2^ daily in culture for 1 and 3 days when compared to respective untreated control cells (Figures 4D,F,H,I), but the expression of *Col1-*α*1* (Figure 4E) was significantly decreased by LLLI of 5.74 J/cm^2^ daily in culture for 3 days.

All 6 genes were expressed at higher levels in chondrocytes cultured for 5 days and the expression of *Acan*, *Col2-*α*1*, *CTNNB1*, and *Sox9* (Figures 4D,F,H,J) was further significantly upregulated (*P* < 0.01) by LLLI of 5.74 J/cm^2^ daily in culture for 5 days while the expression of *Col1-*α*1* and *IL-1*β was significantly (*P* < 0.01) downregulated (Figures 4E,I). The relative level of phenotypic markers plotted using the ratio of LLLI/control amplicons showed increased *Acan* and *Col2-*α*1* and decreased *Col1-*α*1* (Figure 4G).

Category 2: Genes encoding regulators/mediators of the cell death cascade and matrix degradation. LLLI treatments modulated the expression of genes assigned in this group with generated patterns shown in Figure 5 when synergistically treated with the pro-inflammatory cytokine IL-1β.

The LLLI-modulated expression of genes associated with cell death and destruction is summarized in Table 1, showing the overall regulatory trend. Under the lower LLLI treatment (5.74 J/cm^2^ in culture daily for 1 day), the expression of the destructive gene *ADAMTS5* (Figure 5A) was not affected, *Cas-3* (Figure 5B) and *FADD* (Figure 5C) were downregulated, *MMP-13* (Figure 5D) and *TNF-*α (Figure 5E) were unchanged when expressed at a low level, but *TNFR1* (Figure 5F) and *TRADD* (Figure 5G) were upregulated. Most of the genes expressed under the influence of IL-1β were largely unaffected by LLLI of 5.74 J/cm^2^ at day 1, but were either downregulated or unchanged when treated with LLLI of 5.74 J/cm^2^ daily in culture for 3 days (Figure 5 and Table 1). However, the LLLI treatment inhibited the upregulation of *ADAMTS5* (Figure 5A), *Cas-3* (Figure 5B), and *TRADD* (Figure 5G) seen in response to treatment with IL-1β. Furthermore, the expression of these genes was significantly decreased (*P* < 0.01) by LLLI of 5.74 J/cm^2^ daily in culture for 5 days and highly increased by IL-1β, which was prevented by the LLLI treatment (Table 1).

**TABLE 1:** Summary of gene expression in cultured chondrocytes under the regulation of IL-1β and LLLI^∗^.

**Gene**	**1 X IU**	**IL-1β**	**1 X IU + IL-1β**	**3 X IU**	**IL-1β**	**3 X IU + IL-1β**	**5 X IU**	**IL-1β**	**5 X IU + IL-1β**
*ADAMTS5*	1.13	1.91 ↑	2.06	0.88 ↓	2.60 ↑	2.22 ↓	1.13 ↓	7.72 ↑	2.24 ↓
*Cas-3*	0.89 ↓	0.80 ↓	0.78	0.58 ↓	0.88	0.77 ↓	0.42 ↓	0.69 ↑	0.51 ↓
*FADD*	0.46 ↓	1.19 ↑	1.12	0.63 ↓	0.82	0.75	0.45	0.57	0.43 ↓
*MMP-13*	1.30	1.12	1.04	4.87 ↓	8.07	7.50	40.15 ↓	47.73 ↑	34.31 ↓
*TNF-*α	0.94	5.78 ↑	6.68	0.95	15.01 ↑	13.27	3.46 ↓	86.86 ↑	7.15 ↓
*TNFR1*	1.16 ↑	1.11 ↑	1.21	1.01	1.20 ↑	1.13	0.65 ↓	1.37 ↑	0.88 ↓
*TRADD*	1.43 ↑	1.43 ↑	1.76 ↑	1.20 ↓	1.85 ↑	1.59 ↓	1.02 ↓	1.52 ↑	0.87 ↓

*IL-1β profoundly increased the level of “death” mediators assessed and the two matrix degrading proteases, *ADAMTS5* and *MMP-13*, indicating apoptosis and matrix destruction, which were significantly, reversed by 5 X IU LLLI treatment. Arrows “↓” and “↑” show statistically significant down- and up-regulated gene expression, respectively (*P* < 0.01).

### qRT-PCR Verification of LLLI Modulation in Cultured Human C28/I2 Chondrocytes

**FIGURE 6:**
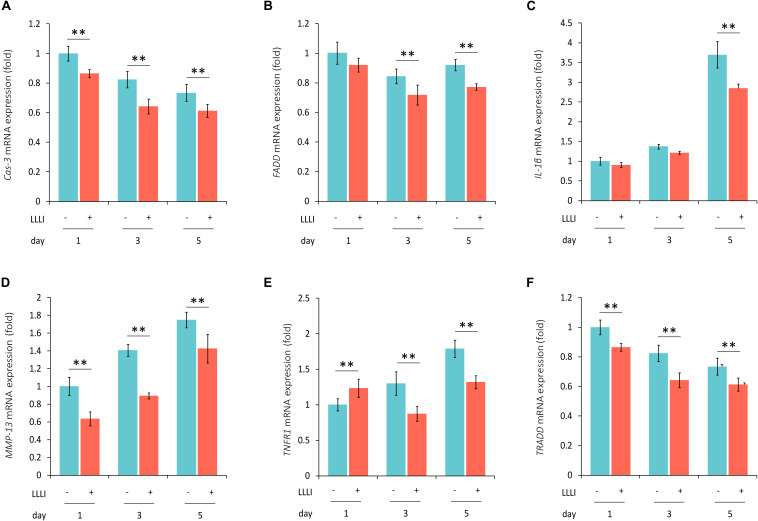
qRT-PCR analysis of the expression of genes associated with cell death and matrix destruction in human C28/I2 chondrocytes. The cells were treated with LLLI of 5.74 J/cm^2^ daily in culture for 1, 3, and 5 days, respectively, followed by further culture for 24 h before harvested for isolation of total RNA (*n* = 3). The expression of 4 genes were significantly (^∗∗^) downregulated by all dosages of LLLI compared to the respective control, i.e., *Cas-3* **(A)**, *MMP-13* **(D)**, *TNFR1* except for an upregulation by 1 X IU **(E)**, and *TRADD* **(F)**. *FADD* expression seemed to be downregulated by all 3 dosages of LLLI, but was not statistically significant for LLLI of 5.74 J/cm^2^ daily in culture for 1 day **(B)**. The expression of *IL-1*β was relatively stable in LLLI of 5.74 J/cm^2^ daily in culture for 1 and 3 days compared to controls. Expression was increased in untreated and treated cells by 5 days with expression significantly (^∗∗^) downregulated by LLLI of 5.74 J/cm^2^ daily in culture for 5 days **(C)**. *GAPDH* was used as an internal reference. The figure was plotted using data from triplicate samples, each with triplicate measurements. Daily LLLI treatment = 5.74 J/cm^2^ of LLLI for 8 min; Error bar = SD; ^∗∗^*P* < 0.01.

The modulation of cellular metabolism by LLLI was also investigated using qRT-PCR in human C28/I2 chondrocytes. The expression of the executioner caspase *Cas-3* was significantly (*P* < 0.01) reduced during cultivation by all 3 doses of LLLI treatment (Figure 6A). Among the examined three key mediators of the TNF/TNFR activated extrinsic cell apoptosis pathway, *FADD* expression was significantly (*P* < 0.01) downregulated by LLLI of 5.74 J/cm^2^ daily in culture for 5 days (Figure 6B). The expression of catabolic gene *IL-1*β was increased in cells cultured for 5 days, which was significantly (*P* < 0.01) counteracted by the LLLI (Figure 6C). Although the collagenase MMP-13 was increasingly expressed as the chondrocytes were cultured for a longer time, it was significantly (*P* < 0.01) decreased by all 3 dosages of LLLI treatments (Figure 6D). The *TNFR1* expression in the chondrocytes was increased by a longer culture time, but was significantly (*P* < 0.01) decreased by LLLI of 5.74 J/cm^2^ daily in culture for 3 and 5 days (Figure 6E). The expression of the death domain partner *TRADD* gene was seemingly decreasing as in 3 periods (days) of cultivation, it was significantly (*P* < 0.01) further reduced by all 3 dosages of LLLI treatments (Figure 6F).

### Determination of Live/Dead Ratio of Cultured Chondrocytes by Double Fluorescent Labeling

**FIGURE 7:**
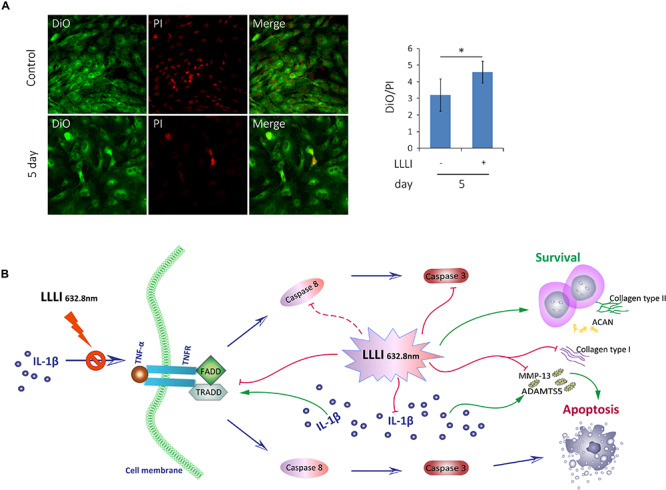
Inhibition of LLLI on apoptosis of cultured rabbit chondrocytes by modulation of the extrinsic apoptosis pathway. **(A)** Chondrocytes in culture were treated with LLLI of 5.74 J/cm^2^ daily in culture for 5 days and labeled by DiO/PI for live/dead cells, which show live cells as green and the dead cells as red fluorescence under CLSM. Both treated and untreated groups showed live cells (green) and dead cells (red) and merged images of the population (left panel). The intensity of fluorescent color was acquired by CLSM using dual channels and the green/red ratio of treated cells was quantitatively compared to that of control cells, showing significantly (^∗^) more live cells than control (right panel). **(B)** Schematic illustration of LLLI-inhibited expression of collagenases *ADAMTS5* and *MMP-13*, and the fibroblastic *Cal1*α*1*; the IL-1β-induced cytokine *TNF-*α and its receptor *TNFR1*, the association of which triggers the recruitment of the death domain (DD) together with TNF receptor-associated death domain (*TRADD*), Fas-associated death domain (*FADD*) proteins to form the death inducing signaling complex (DISC). The DISC activates the initiating caspases, including the known initiator caspase-8 (unexamined in this study and indicated by a dotted line), which, in turn, activates executioner caspases, such as caspase-3, to destroy cellular substrates causing cell apoptosis. Scaled bar (—) represents 100 μm. Daily LLLI treatment = 5.74 J/cm^2^ of LLLI for 8 min; Error bar = SD; ^∗^*P* < 0.05.

The cultured chondrocytes were labeled with the membrane lipophilic fluorescent dye DiO in green and the nucleic acid dye PI in red, which represent live and dead cells, respectively. The labeled cells were observed and analyzed using dual channels of CLSM. The acquired images (Figure 7A, left) showed live cells in green (left) and dead cells in red (middle) and the merged image showing the mixture of live/dead cells (right). More live cells were visible in the chondrocytes treated by LLLI of 5.74 J/cm^2^ daily in culture for 5 days (bottom panel) than control cells (top panel), which was further quantitatively illustrated by the ratio of live/dead labels (Figure 7A, right) with statistical significance (*P* < 0.05).

## Discussion

Viable chondrocytes are essential for both physiologic remodeling and reparative regeneration in cartilage. The latter requires expanding the cells initially isolated from limited biopsy cartilage specimens *ex vivo* (Gibson et al., 2006; Brittberg, 2008) for at least 4 passages (Darling and Athanasiou, 2005). These cells secrete physiologically balanced matrix proteins for the restoration of functional cartilage (Martin et al., 2004). However, passaged chondrocytes change their gene expression profiles (Lin et al., 2008) leading to dedifferentiation (Benya and Shaffer, 1982; Stokes et al., 2001; Hsu et al., 2002; Darling and Athanasiou, 2005; Frohlich et al., 2007). Dedifferentiated chondrocytes generate fibroblastic cartilage incapable of functional repair, which provokes efforts to retain the chondrogenic phenotype either by optimizing culture conditions (Takahashi et al., 2007) or re-differentiating experimentally (Taylor et al., 2015) with little progression achieved in the field. This study examines the biostimulatory effects of LLLI on passaged chondrocytes for obtaining large numbers of differentiated chondrocytes.

Light has long been used for therapeutic treatments, leading to the discovery of suitable light sources, including the latest lasers (Monici and Cialdai, 2012) developed for modern phototherapy (McDonagh, 2001; Hockberger, 2002). The key of LLLT is to modulate metabolic activity of cells within tissues or in isolation to allow physiological changes to occur in the target cells (Pinheiro et al., 2002) in a “biphasic dosage response” fashion (Calabrese, 2005). LLLT shows its effectiveness for a variety of treatments, including treatments for tissue and cells (as summarized in the Introduction), and limited approaches in chondrogenesis. The LLLT studies look to identify a threshold of irradiation intensity for maximum biostimulation in target cells of interest by optimizing wavelengths, power densities, and exposure time. LLLT settings for effectively enhancing proliferation in various cells used in the past 80 years fell into the energy density range of 0.5–4.0 J/cm^2^ and a visible spectrum between 600 and 700 nm (Alghamdi et al., 2012). The use of LLLT on chondrocytes started by treating cartilage and assessing metabolic changes in cells embedded in the tissue. Using LLLI of blue laser (405 nm) at 100 mW/cm^2^, the expression of chondrogenic genes were stimulated in prechondrogenic ATDC5 cells (Kushibiki et al., 2010).

Cell culture is an essential element to address demand for cell-based therapies in cartilage repair. Laser treatment (780 nm, 2500 mW) on human chondrocytes in culture for 5 days showed “good” viability and metabolism of calcium and alkaline phosphate without toxicity (Morrone et al., 2000). Rabbit and human chondrocytes in culture treated for 5 days by laser (300 J and 1 W) with varying frequencies (hertz) showed increased cell proliferation that was maintained for five additional days (Torricelli et al., 2001). Chondrocytes treated with LLLI (658, 785, and 830 nm with 10–70 mJ/cm^2^) in culture with 2 and 5% PBS, but 0 and 10% FBS, responded with significantly promoted proliferation (Zheng et al., 2015). Nevertheless, chondrocytes need to be expanded for several passages to meet the therapeutic requirement (Darling and Athanasiou, 2005).

In this study, we used a LLLI with 632.8 nm and 5.74 J/cm^2^ intensity in similar culture conditions as previously reported, and achieved stimulated proliferation (Figure 1) in cultured chondrocytes. As assessed by MTS, chondrocytes treated with two doses of LLLI (output power of 12 mW over 0.91 cm^2^ daily for 1 and 3 days) in a time course showed “slightly” but significantly (*P* < 0.05) increased proliferation (Figures 1A,B). This was comparable to previous results by MTT/MTS and supported by the observation of more live cells in LLLI treated chondrocytes in culture from the live/dead fluorescent staining assay (Figure 7A). Moreover, the “best” response in both LLLI intensities was achieved by a treatment of 8 min. Based on the enhanced proliferation by MTS and the promoted GAG synthesis and release by chondrocytes that was modulated and maintained by LLLI of 5.74 J/cm^2^ daily in culture for 5 days and for 6 days with further culture up to 9 days (Figure 1C), the LLLI treatment regime was adopted for SEM analysis in the search for supportive cytomorphological evidence, particularly for assessing cellular/subcellular changes in cells post LLLI treatment, where a higher GAG level was maintained (between day 6 and day 12) in the treated group than in the control. The SEM results provided signs of LLLI-stimulated cellular metabolism supportive of the active GAG secretion in the cultured chondrocytes (Figure 2), alongside increased nucleic acid synthesis (Figure 3) and modulated expression of genes and proteins involved in chondrogenesis, cell apoptosis, and destruction.

Chondrocytic matrix proteins, such as ACAN and COL I and II, are major products synthesized and released under a tight regulation of various growth/signaling factors, such as the chondrogenic master transcription factor Sox9 (Bi et al., 1999; Akiyama et al., 2002), which directly binds to activate genes coding for COL II, COL XI, and ACAN (Bi et al., 1999), and also interacts with β-catenin, a central player of the canonical Wnt signaling pathway (Stamos and Weis, 2013) to modulate chondrocyte growth (Akiyama et al., 2004). The pro-inflammatory cytokine IL-1β represents a group of cytokines that profoundly affects cell growth and cell fate determination (Kapoor et al., 2011) by activating apoptosis (Qin et al., 2012). The results of this study elucidated that the expression of *Acan*, *Col2-*α*1*, *CTNNB1*, and *Sox9* genes was upregulated in a synergistic manner (Figures 4D,F,H,J) while adverse genes of *Col1-*α*1* and *IL-1*β were downregulated (Figures 4E,I), particularly shown in cultured chondrocytes treated by LLLI of 5.74 J/cm^2^ daily in culture for 5 days. Intriguingly, LLLI restrained the expression of *Col1-*α*1* (Figure 4E) and enhanced *Col2-*α*1* (Figure 4F), which was also confirmed at protein levels (Figures 4A–C) indicating maintenance of the chondrocytic phenotype together with increased *Acan* (Figures 4A,G), which opposed previous reports of dedifferentiation in passaged chondrocytes *ex vivo* (Darling and Athanasiou, 2005; Frohlich et al., 2007). These results show LLLI is capable of simultaneously stimulating cell growth and modulating cellular metabolism toward generation of large numbers of chondrocytes *in vitro* with differentiated phenotype, essentially relevant to functional repair of articular cartilage by tissue engineering.

**FIGURE 8:**
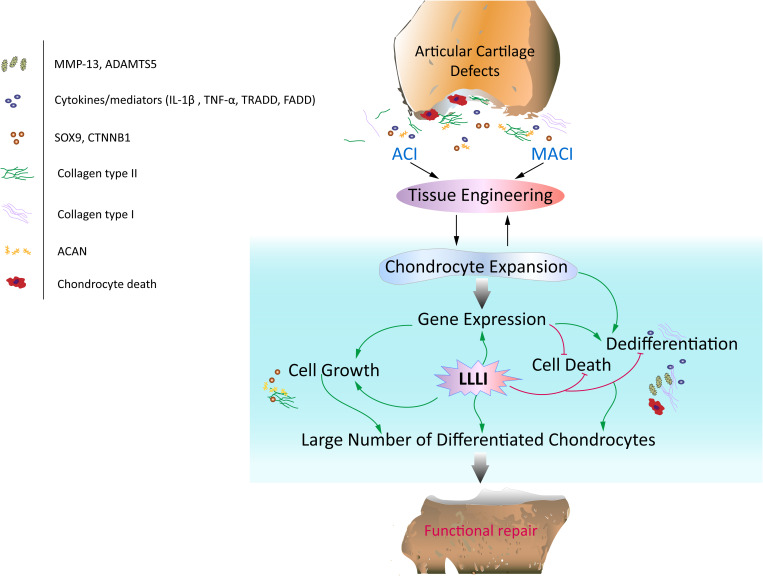
Schematic illustration of the potential of LLLI to synergize with currently effective clinical cell-based therapies for articular cartilage defects. Autologous chondrocyte implantation (ACI) and matrix-induced autologous chondrocyte implantation (MACI) procedures rely on generation of a large number of chondrocytes expanded *ex vivo*, which secrete matrix component proteins in a homeostatic manner for regeneration of hyaline cartilage essential for restoration of functional joints. *In vitro* expansion of chondrocytes by conventional cultivation usually results in dedifferentiation to fibroblastic chondrocytes less capable of regenerating functional cartilage. Laser irradiation biostimulation was assessed for its capacity to generate large numbers of chondrocytes in culture potentially useful in functional repair of defected cartilage. The doses of low level laser irradiation (LLLI) applied in this study stimulated the expression of chondrogenic genes such as *Sox9*, *CTNNB1*, *Acan* and *Col2*α*1*, and inhibited “destructive” factors/mediators including the cytokines *IL-1*β and *TNF-*α, members of the death cascade, matrix degrading enzymes and the fibroblastic *Cal1*α*1* leading to increased proliferation and reduced cell death and the generation of large numbers of cultured chondrocytes with high viability and a differentiated phenotype suitable for therapeutic applications in tissue engineering.

Generation of usable, functional chondrocytes could result from strengthened cell growth and enhanced viability or reduced cell death. The pro-inflammatory cytokine IL-1β-induced cells with increased expression of *TNF-*α which associates with its surface receptor TNFR (Saperstein et al., 2009) to initiate the formation of the death inducing signaling complex (DISC) (Riedl and Salvesen, 2007). DISCs recruit death domain mediators of FADD and TRADD, which triggers the activation of cysteine-aspartic proteases caspase-8 and -3 (Qin et al., 2012) in the extrinsic apoptosis signaling pathway (Riedl and Salvesen, 2007). The effect of LLLI on TNF/TNFR-mediated cell death cascade was checked in cultured chondrocytes in this study. IL-1β was shown to increase the expression of these apoptotic mediators in cultured cells, including increased *TNF-*α, regardless of the duration of culture and intensity of LLLI treatments (Figure 5E). The addition of IL-1β to the medium, together with its stimulated *TNF-*α, upregulated expression of cellular apoptotic mediators/factors (Figure 5 and Table 1), including *Cas-3* (Figure 5B). The action of IL-1β and its consequences were overwhelmingly counteracted by LLLI, as particularly shown in chondrocytes cultured for 5 days, where IL-1β-induced gene expression of all destructive factors were offset by LLLI of 5.74 J/cm^2^ daily in culture for 5 days (Figure 5 and Table 1) except for unchanged *FADD* levels (Figure 5C). It is worth noting that the expression of genes coding for the initiating apoptotic cytokine *TNF-*α (Figure 5E) and the two matrix degrading proteases *ADAMTS5* (Figure 5A) and *MMP-13* (Figure 5D) in the cultured chondrocytes were also highly increased and then significantly counteracted by LLLI of 5.74 J/cm^2^ daily in culture for 5 days in both IL-1β-treated and untreated groups. As detected by Western blot, the executioner caspase, Cas-3 (Figure 5H, left) and the matrix destructive collagenase MMP-13 (Figure 5H, right) were significantly reduced by LLLI, highly increased by IL-1β, and then offset by LLLI treatments. Additionally, the gene expression pattern, implicating that LLLI-modulated genes involved in cell apoptosis, was also reflected at the protein level. The expression of death mediator genes (*Cas-3*, *FADD*, *TNFR1* and *TRADD*), *IL-1*β, and *MMP-13* was also modulated by LLLI in human C28/I2 chondrocytes in culture in a similar pattern and extent to that in rabbit cells (Figure 6), strongly validating the mechanism of LLLI regulation across species and the potential to enable generation of functional human chondrocytes. Further, as demonstrated by live/dead fluorescent staining experiments, cultured chondrocytes treated by LLLI of 5.74 J/cm^2^ daily in culture for 5 days had a significantly (*P* < 0.05) higher live/dead ratio than the untreated control (Figure 7A), suggesting inhibited cell death, at least partially, through blocking the extrinsic cell apoptosis pathway (Figure 7B). The strengthened cell viability and metabolism, inhibited apoptotic mediators/regulators of the apoptosis signaling pathway and matrix degrading proteases, and rectified differentiated phenotype in cultured chondrocytes in monolayer by LLLI in the current study could benefit a strategy for generating large number of viable chondrocytes with differentiated phenotype potentially applicable in cell-based therapies for cartilage regeneration (as proposed in Figure 8).

## Conclusion

Despite the “mild” increase in LLLI promoted proliferation showed by MTS assay, the current study provided clear cytomorphological evidence of enhanced cell growth and anabolic synthesis, and comprehensively investigated the modulation of LLLI on the expression of chondrogenic and apoptotic/destructive genes in cultured chondrocytes. Treatment by LLLI showed patterns of enhanced chondrogenesis and reduced apoptosis, suggesting the possibility of production of the large numbers of cultured chondrocytes required for cell-based cartilage repair therapies. Moreover, the LLLI-modulated chondrocytes in culture maintained their differentiated phenotype on expansion, highlighting their potential for application in cartilage lesion repair as a cell-based therapy.

## Data Availability Statement

All datasets generated for this study are included in the article/Supplementary Material.

## Ethics Statement

The animal study was reviewed and approved by the governing authority of Guangzhou Red Cross Hospital, Jinan University School of Medicine (SHYYLS 2017-004-01 and SHYYLS 2019-040-01).

## Author Contributions

XY, SL, and SC initiated and supervised the project, and designed laser irradiation scheme with TL. WZ, HL, PL, and SY performed animal manipulation, cartilage tissue collection under the supervision of XY. XY, SL, and HL carried out cell culture and laser irradiation. XY, SC, and WZ conducted molecular experiments. XY, SC, TL, SL, and WZ participated in data analysis and interpretation. SC and XY wrote the manuscript. SC made revisions of the manuscript, which was approved by all authors for publication.

## Conflict of Interest

The authors declare that the research was conducted in the absence of any commercial or financial relationships that could be construed as a potential conflict of interest.
