# Supplementary material for: Promoted Viability and Differentiated Phenotype of Cultured Chondrocytes With Low Level Laser Irradiation Potentiate Efficacious Cells for Therapeutics
Source: Front Bioeng Biotechnol. 2020 May 29;8:468. doi: 10.3389/fbioe.2020.00468 (PMC7272569; doi:10.3389/fbioe.2020.00468)
Supplement: Supplementary file 1 [file Table_1.pdf]

Table 1. Primers designed for qRT-PCR analysis*

Target genes	Acc. No.	Sequence (5'---3')	Amplicon size (bp)
**Rabbit chondrocyte growth and phenotype related genes**			
ACAN	XM_002723376	FWD: GGATGGACACCCCCTACAA	
		REV: AGGGGACGTCATTCCACTC	122
Col1-α1	XM_017348831.1	FWD: TGCCCAGAAGAACTGGTACA	
		REV: AAGCCATCGGTCATGCTCTC	81
Col2-α1	NM_001195671	FWD: GACGACATAATCTGTGAAGACACC	
		REV: GTTCTCCTTTCTGCCCCTTTG	133
CTNNB1	XM_002713075	FWD: ATGACTCGAGCTCAGAGGGT	
		REV: TGCGTGTTTCAGCATTTGGG	144
IL-1β	NM_001082201.1	FWD: GGTGTTGTCTGGCACGTATG	
		REV: GGCCACAGGTATCTTGTCGT	124
Sox9	XM_002719499	FWD: CTGGAGACTGCTGAACGAGAG	
		REV: GGTACTTGTAGTCCGGGTGGT	98
**Rabbit cell death and destructive factors**			
ADAMTS5	XM_002716775.3	FWD: CCATGAGGAGCACTACGACG	
		REV: ATATGGTCCCAACGTCTGCC	101
Cas-3	NM_001082117.1	FWD: GCATATTCCACAGCACCTGG	
		REV: TGTCGCTACCTTCCGGTTCA	150
FADD	XM_008253090.2	FWD: GAAGTCACAGCTGGAATGGC	
		REV: CCTGAACGCGTGGTTTCTTT	95
MMP-13	NM_001082037.1	FWD: CCTACACCGGCAAGAGTCAC	
		REV: GGTGTTTAGGGTTGGGGTCT	99
TNF-a	NM_001082263.1	FWD: ACAAGCCTCTAGCCCACGTA	
		REV: AGATGAGGTACAGCCCGTCG	147
TNFR1	XM_002712835.3	FWD: GGAAGTGTGAAAACGGCACC	
		REV: TGTCATGGTCCACTGTGCAA	123
TRADD	XM_008257435.2	FWD: GTCGGACATCTACGCACAGC	
		REV: ATCTTGAGCATTTGCACCACG	108
**Rabbit internal control-house-keeping gene**			
GAPDH	NM_001082253	FWD: AGGGCTGCTTTTAACTCTGG	
		REV: ATGACCAGCTTCCCGTTCT	149
**Human death and destructive genes**			
Cas-3	XM_011532301.1	FWD: CCATTGTTTGTGGGAAATCG	
		REV: GGAGGTGAAGGTGGAACTGG	152
FADD	NM_003824.3	FWD: CTGGCTCGTCAGCTCAAAGT	
		REV: GTTGCGTTCTCCTTCTCTGTG	128
IL-1β	NM_000576.3	FWD: GCCCTAAACAGATGAAGTGCTC	
		REV: GAACCAGCATCTTCCTCAG	156
MMP-13	NM_002427.3	FWD: TTGAGCTGGACTCATTGTCG	

		REV: CGCGAGATTTGTAGGATGGT	126
TNFR1	NM_001346091.1	FWD: CCATTGTTTGTGGGAAATCG	
		REV: GGAGGTGAAGGTGGAACTGG	152
TRADD	XM_005256213.3	FWD: TTTGAGTTGCATCCTAGCCC	
		REV: GCACTTCAGATTTCGCAGC	88
**Human internal control-house-keeping gene**			
GAPDH	NM_001289746.1	FWD: ACACCCACTCCTCCACCTTT	
		REV: TTACTCCTTGGAGGCCATGT	143

*These genes, from both rabbit and human, markedly fall into 2 categories, chondrocyte growth and phenotype defining genes, and cell death and destructive factors. *GAPDH* serves as an internal reference gene for quantitation.
